# Editorial: Strategic molecular biomarkers and microRNAs in cancer

**DOI:** 10.3389/fonc.2022.1031349

**Published:** 2022-10-12

**Authors:** César López-Camarillo, Ondrej Slaby, Macrina Beatriz Silva-Cázares

**Affiliations:** ^1^ Posgrado en Ciencias Genómicas, Universidad Autónoma de la Ciudad de México, Ciudad de Mexico, Mexico; ^2^ Molecular Oncology II-Solid Cancer Central European Institute of Technology, Brno, Czechia; ^3^ Coordinación Académica Región Altiplano, Universidad Autónoma de San Luis Potosí, Matehuala, Mexico

**Keywords:** Molecular Biomarker, MicroRNAs, cancer, gene, expression

## Introduction

Breast cancer is a highly heterogeneous disease. Throughout the last few years, various therapies emerged in the era of cancer genomics. Advances in breast cancer have been made at the molecular and genomic levels, which facilitate us in identifying new prognostic markers and therapeutic targets. microRNAs (miRNAs) are implicated in carcinogenesis and their expression supply potential markers for cancer detection and progression. The biomarkers should predict not only prognosis, but also the response to therapies.

There are several Research Topics mentioning here. In 2001, a Research Topic titled “*Non–coding RNA genes and the modern RNA world*” was published in “Nature Review Genetics” (1). In 2009, Shi (2009) gave a review of microRNA expression and its implications for the diagnosis and therapeutic strategies of breast cancer (2). In 2019, Frontiers in Oncology published another Research Topic titled “*From “Junk DNA” to Clinically Relevant Tools for Cancer Diagnosis, Staging, and Tailored Therapies: The Incredible Case of Non-Coding RNAs*” (3) Recently, there is progress in research on molecular biomarkers and microRNAs in cancer, for example, noncoding RNA (ncRNAs) (4).

The present Research Topic “*Strategic Molecular Biomarkers and MicroRNAs in Cancer*” aimed at widening the knowledge on novel biomarkers and microRNAs in cancer biology and therapy emphasizing interdisciplinary contributions. The issue currently includes 9 manuscripts on the analysis of miRNAs expression in cancer cells, usefulness of cancer gene panel tests and miRNA analysis in histopathological diagnosis and functions emphasizing their contributions as important molecular markers for cancer diagnosis. The studies presented in the Special Issue arise from diverse fields across biology molecular, oncogenomic, and clinical cancer.

## Analysis of miRNAs expression in cancer cells

Micro-RNAs (miRNAs) are currently used as cancer biomarkers. Rajthala et al., investigated miR-138 dysregulation in cancer-associated fibroblasts (CAFs) in oral squamous cell carcinoma (OSCC) and its effects. The expression of miR-138 showed marked heterogeneity in both OSCC tissues and cultured fibroblasts. Ectopic miR-138 expression reduced fibroblasts’ motility and collagen contraction ability and suppressed invasion of suprajacent OSCC cells, while its inhibition resulted in the opposite outcome. In conclusion, this study supports a tumor-suppressive role for miR-138 in OSCC while expressed in stromal fibroblasts, despite its heterogeneous expression. The miR-15b-5p is encoded by MIR15B gene which participates in the pathogenesis of several cancers as well as non-malignant conditions, such as abdominal aortic aneurysm, Alzheimer’s and Parkinson’s, cerebral ischemia reperfusion injury, coronary artery disease, dexamethasone induced steatosis, diabetic complications and doxorubicin-induced cardiotoxicity. Dysregulation of miR-15b-5p in clinical samples has been associated with poor outcome in different kinds of cancers. In this review, the authors discuss the role of miR-15b-5p in malignant and non-malignant conditions (Ghafouri-Fard et al., 2022).

## Usefulness of cancer gene panel test and miRNAs analysis in histopathological diagnosis

MicroRNAs (miRNAs) are endogenous, noncoding, single-stranded RNAs that regulate gene expression, a characteristic that confers the potential for identifying malignancy (5). Dai et al. describe an original research article, of the fusions of receptor tyrosine kinase (RTK) involving anaplastic lymphoma kinase (ALK), c-ros oncogene 1 (ROS1), and neurotrophic receptor tyrosine kinase (NTRK) represent the potential targets of therapeutic intervention for various types of solid tumors. These results may provide genomic information for the personalized clinical management of solid tumor patients with ALK, ROS1, and NTRK fusions in the era of precision medicine. In other hand, Zhang et al., identified a prognostic signature composed of enhancer RNA-regulated genes (eRGs) for hepatocellular carcinoma (HCC). The findings of this study have significant practical implications in terms of providing a deeper insight into the investigation of pathogenesis of HCC, optimizing individualized treatment, and improving the prognosis of HCC patients.

## Functions of miRNAs in relation to important molecular markers for cancer diagnosis

Salinas-Vera et al., present a review about microRNAs regulation of cancer hallmarks in 3D cell cultures from different types of cancers. The authors discuss the utilization of different types of 3D culture models including spheroids, organotypic models and patient-derived organoids in gynecologic cancers research, as well as its potential applications in oncological research mainly for screening drugs with major physiological and clinical relevance. Huang et al., present epidemiological evidence between variants in matrix metalloproteinases-2, -7, and -9 and cancer risk. Their findings support the relations between variants of MMP-2, MMP-7, and MMP-9 and various cancers risk, demonstrating the credibility of these relations and offer valuable data to design future research to assess variants in MMP factors for cancer risk.

## Perspectives

In conclusion, investigations of molecular biomarkers and microRNAs continue to be essential in the development of new strategies that produce more successful treatments in human cancers. It is clear that target molecules of miRNAs are useful as molecular markers for cancer, and research to clarify the functions of miRNAs and their target molecules has an important role in the treatment of cancer. This Research Topic could contribute to the actual efforts focused in the search of novel biomarkers and microRNAs with potential applications on oncology research and therapy.

## Author contributions

All authors listed have made a substantial, direct, and intellectual contribution to the work and approved it for publication.

## Acknowledgments

We deeply thank all the authors and reviewers who have participated in this Research Topic. We also thank to the editorial team at Frontiers for their invaluable support.

## Conflict of interest

The authors declare that the research was conducted in the absence of any commercial or financial relationships that could be construed as a potential conflict of interest.

## Publisher’s note

All claims expressed in this article are solely those of the authors and do not necessarily represent those of their affiliated organizations, or those of the publisher, the editors and the reviewers. Any product that may be evaluated in this article, or claim that may be made by its manufacturer, is not guaranteed or endorsed by the publisher.

## References

[1] Eddy SR. Non-coding RNA genes and the modern RNA world. Nat Rev Genet (2001) 2(12):919–29. doi: 10.1038/35103511 11733745

[2] Shi M Guo N. MicroRNA expression and its implications for the diagnosis and therapeutic strategies of breast cancer. Cancer Treat Rev (2009) 35(4):328–34. doi: 10.1016/J.CTRV.2008.12.002 19171434

[3] Iorio MV Palmieri D. Editorial: From “junk DNA” to clinically relevant tools for cancer diagnosis, staging, and tailored therapies: From incredible case of non-coding RNAs. Front Oncol (2019) 9:389(MAY). doi: 10.3389/FONC.2019.00389 31139571 PMC6527587

[4] Zhou JG Huang Z Slaby O Navarro A. Editorial: The role of ncRNAs in solid tumors prognosis: From laboratory to clinical utility. Front Oncol (2020) 10:631316. doi: 10.3389/FONC.2020.631316 33604302 PMC7884852

[5] Ciarletto AM Narick C Malchoff CD Massoll NA Labourier E Haugh K. Analytical and clinical validation of pairwise microRNA expression analysis to identify medullary thyroid cancer in thyroid fine-needle aspiration samples. Cancer cytopathology (2021) 129(3):239–49. doi: 10.1002/CNCY.22365 PMC7984450 33017868

